# Case completeness in the Norwegian Cardiac Arrest Registry

**DOI:** 10.1016/j.resplu.2021.100182

**Published:** 2021-11-14

**Authors:** Kristin Alm-Kruse, Ingvild Tjelmeland, Håvard Kongsgård, Rune Kvåle, Jo Kramer-Johansen

**Affiliations:** a Department of Research and Development, Division of Emergencies and Critical Care, Oslo University Hospital, Oslo, Norway; b Faculty of Medicine, Institute of Clinical Medicine, University of Oslo, Oslo, Norway; c Division of Prehospital Services, Oslo University Hospital, Oslo, Norway; d Institute for Emergency Medicine, University Hospital Schleswig-Holstein, Kiel, Germany; e Department of Oncology, Haukeland University Hospital, Bergen, Norway; f Department of Health Registry Research and Development, National Institute of Public Health, Bergen, Norway

## Abstract

**Introduction:**

This study aimed to assess the case completeness of out-of-hospital cardiac arrests (OHCA) in the Norwegian Cardiac Arrest Registry (NorCAR) and describe the differences between the registered and missing patients identified from the case-control assessment.

**Methods:**

We identified the relevant patients in the Norwegian Patient Registry and the Norwegian Cause of Death Registry and compared them with the patients in NorCAR. Data processors used patient records to confirm if the potential cardiac arrest cases met the inclusion criteria in NorCAR.

**Results:**

Between 2015 and 2017, 8612 OHCA patients were registered in NorCAR. Through the Patient Registry and the Cause of Death Registry we identified 11,114 potential OHCA patients, 3469 of these were already registered in NorCAR. After evaluating the patient records for the remaining 7645 patients, we found 344 patients (4%), were eligible for inclusion in NorCAR, giving a case completeness of 96%. The registered and missing patients were similar in age and gender distribution. Initial shockable rhythm and presumed cause were also comparable. However, the missing patients more frequently achieved return of spontaneous circulation, were more often transported to hospital, and had higher survival rates. The already registered patients had more key variables registered than the missing patients.

**Conclusion:**

Our results indicate high case completeness in NorCAR. The missing patients were too few to introduce significant changes in the distribution of patient characteristics, indicating that NorCAR is representative of the Norwegian OHCA population.

## Introduction

The primary purpose of a health registry is to promote health, prevent disease and injury and improve health care services through quality improvement projects.^1^ To fulfil this purpose, registries need to capture a high and representative proportion of the relevant patients in the population.^2^ The incidence of reported emergency medical services (EMS)-treated out-of-hospital cardiac arrest (OHCA) varies,^3^ a recent study from 28 European countries reported it to be between 27 and 91/100,000 inhabitants.^4^ The difference might be due to variations in actual incidence but also differences in case definitions or the completeness of cases. International consensus on inclusion criteria for cardiac arrest registries should enable comparison of results between studies and registries.2., 5. However, interpretation of definitions and reporting has been found to vary both within and between registries.6., 7. To ensure the validity of results from registries, both data entries and case completeness need rigid quality control.^2^

Few cardiac arrest registries report on their case completeness. Registries for in-hospital cardiac arrest report completeness of between 33% and 78%.8., 9. Registries for OHCA have found case completeness between 75% and 82%.10., 11. The proportion of missing registrations in these studies supports the need for case completeness assessments in cardiac arrest registries.

The main objective of this study was to assess the case completeness of OHCA in the Norwegian Cardiac Arrest Registry (NorCAR). Secondary objectives were to identify differences between the registered and missing cases and evaluate the completeness of key variables for both groups.

## Methods

The target population was all OHCA in Norway ≥ 18 years of age, in 2015-2017. We used data from the Norwegian Patient Registry (Patient Registry) and the Norwegian Cause of Death Registry (Cause of Death Registry) to assess case completeness.

We included cardiac arrest and resuscitation codes in the case-control. Conditions that indirectly could lead to cardiac arrests like trauma, drowning, hypothermia, etc. were not included.

### Norwegian Cardiac Arrest Registry

The Norwegian Cardiovascular Disease Registry included NorCAR as a national quality registry in 2013, making cardiac arrest a reportable condition. All 18 health trusts with ambulance services reported data by May 2016. Unconscious patients, not breathing normally and receiving resuscitation efforts, such as cardiopulmonary resuscitation (CPR) or defibrillation, are included in the registry, regardless of who provides the resuscitation. The registry contains both prehospital and in-hospital cardiac arrests, but the objective for this study was the completeness of OHCA registrations.^12^

Reporting to NorCAR is initiated by ambulance personnel after attending a cardiac arrest. Trained data processors, primarily nurses and paramedics in active EMS duty, enter the event data. They are encouraged to audit multiple sources for missing patient cases; including emergency medical communication centers (EMCC) records, ambulance and air-ambulance logbooks, electronic hospital records, and administrative data.

### Norwegian Patient Registry

The Patient Registry is a national administrative registry that includes demographic data and medical information about hospitalisation and treatment. The Patient Registry comprises data on an individual level and covers all public specialist healthcare services in Norway, including private institutions and medical specialists contracted to the regional health authorities.13., 14.

From the Patient Registry, we included cases classified as cardiac arrest and resuscitation based on International Statistical Classification of Diseases and Related Health Problems, 10^th^ edition (ICD-10) diagnoses, the Norwegian Classification of Medical Procedures (NCMP) and the Norwegian Classification of Surgical Procedures (NCSP), as well as the dates of admission and discharge for each encounter with a specialist health service (Table 1).

**Table 1:** Source, name and quantity of data used to assess case completeness in the Norwegian Cardiac Arrest Registry.

Source of data	Name	Controlled n = 9389^a^ n (%)	Confirmed n = 416^a^ n (%)	% confirmed
Cause of Death Registry	Underlying cause of death; cardiac arrest	4626 (49)	122 (29)	2.6
Patient Registry ICD-10	I49.0 Ventricular fibrillation and flutter	1642 (18)	67 (16)	4.1
Patient Registry ICD-10	I46.0 Cardiac arrest with successful resuscitation	1422 (15)	135 (33)	9.4
Patient Registry ICD-10	I46.9 Cardiac arrest, cause unspecified	981 (10)	68 (16)	6.9
Patient Registry ICD-10	I46.1 Sudden cardiac death	187 (2)	8 (2)	4.2
Patient Registry NSMP	WDAB80 Closed chest compressions	514 (6)	16 (4)	3.1
Patient Registry NMCP	FYAB81 Open chest compressions	17 (0)	0 (0)	0.0

Abbreviations: Cause of Death Registry, the Norwegian Cause of Death Registry; ICD-10 – International Statistical Classification of Diseases and Related Health Problems, 10^th^ edition; NCMP, Norwegian Classification of Medical Procedures; NCSP, Norwegian Classification of Surgical Procedures; Patient Registry, the Norwegian Patient Registry.

a The unit in the table is episodes. Each patient may be registered with more than one episode.

### Norwegian Cause of Death Registry

The Cause of Death Registry issues the official statistics for Norway on causes of death. This registry contains information on deaths and causes of death in Norway from 1951 until today. It is mandatory to report all deaths to the registry, including Norwegian residents who die abroad.^15^

From the Cause of Death Registry, we retrieved information about the date and location of all persons registered with cardiac arrest as the underlying cause of death (ICD-10) (Table 1).

### Case Assessment

All Norwegian citizens and permanent residents have a unique personal identification number, enabling the linkage of individual data between registries. Patients without valid personal identification numbers were excluded from the study. About two-thirds of OHCA patients die before hospital admission and may be registered in the Cause of Death Registry, with cardiac arrest as the presumed or verified cause of death. The remaining one-third of OHCA patients survive to hospital admission and are included in the Patient Registry.^12^ However, neither the Cause of Death Registry nor the Patient Registry distinguishes between cardiac arrests occurring before or after hospital admission.

Data from the Patient Registry were compared with patients registered in NorCAR as transported to a hospital after OHCA. Data from the Cause of Death Registry were compared with patients registered as dead within 30 days of OHCA in NorCAR. Data were compared on a case-by-case basis within a pragmatic timeframe of ± seven days for the Cause of Death Registry. The timeframe for the Patient Registry was -14/+ 90 days to allow for inaccurate registrations and lengthy hospital stays after the index event. Patients found in NorCAR within the timeframe were accepted as already registered, while patients outside were reviewed from their patient records.

First we manually reviewed electronic EMCC dispatch reports. The case was considered not to be OHCA if no contact had been made with the EMCC within a reasonable time interval of the event. If there had been any contact and the dispatch journals did not confirm a cardiac arrest, the ambulance and/or hospital journals were reviewed. To ensure a uniform process, a NorCAR representative performed the review together with a local data processor. Patients that met the inclusion criteria were added to NorCAR.

### Completeness of Key Variables

The key variables in NorCAR are; “presumed cause of arrest”, “CPR by EMS personnel”, “location of cardiac arrest”, “witnessed status”, “bystander CPR” and “sustained ROSC”. If a variable had the value “unknown” or “missing”, it was defined as incomplete. We compared the proportion of complete registrations between the registered and missing patients.

### Statistical Methods

The comparisons between the groups were performed by Fisher’s exact test for the categorical data and the Mann-Whitney *U* test for the continuous and skewed data. Chi-square was used to look at trend over time for categorical data. A *p*-value of < 0.05 was considered statistically significant. The analysis was performed with the Statistical Package for Social Sciences (IBM SPSS Statistics, version 26.0, NY, USA). Missing registrations from health trusts that did not contribute data at the time of the arrest were excluded from the analysis.

### Ethics

The Regional Committee for Medical and Health Research Ethics (2017/1263) and the local data integrity officer (17/16621) at Oslo University Hospital approved the study. The steering committee for NorCAR approved data disclosure to this study. A data protection impact assessment was performed in cooperation with a local data integrity officer. The Norwegian Institute of Public Health approved the data extraction based on this assessment (18-0121 for the Patient Registry and 18-0488 for the Cause of Death Registry).

## Results

**Fig. 1:**
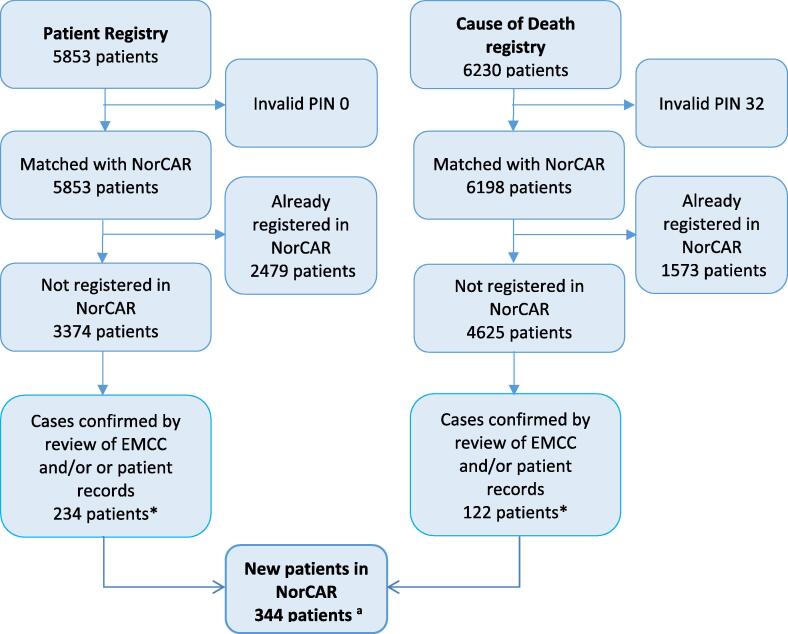
The audit of case completeness in the Norwegian Cardiac Arrest Registry (NorCAR). Data from the Norwegian Patient Registry and the Norwegian Cause of Death Registry was used to identify potential cardiac arrest cases. The unit is individual patients. Patients without valid personal identification numbers (PIN) were excluded from the case-control;NorCAR had eight patients without valid PINs. Abbreviation: EMCC – Emergency Medical Communication Centres ^a^ Some patients were in both the Patient Registry and the Cause of Death Registry, creating a smaller total of new patients in NorCAR.

**Fig. 2:**
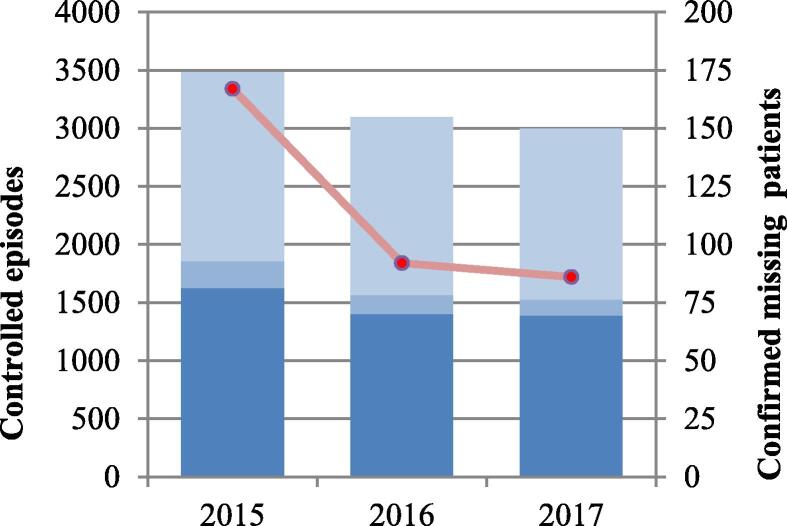
Controlled episodes and missing patients in the Norwegian Cardiac Arrest Registry. Potential cases of cardiac arrest were identified in the Norwegian Patient Registry and the Norwegian Cause of Death Registry. Patient records were reviewed to confirm missing patients.Dark blue bars: ICD-10 diagnoses from the Patient Registry; blue bars: procedures from the Patient Registry; light blue bars: ICD-10 diagnoses from the Cause of Death Registry; red line: number of missing patients in the Cardiac Arrest Registry (right axis).

During 2015-2017, NorCAR registered 9869 patients with cardiac arrest. Of these, 8612 patients had OHCAs, and 1257 had in-hospital cardiac arrests. During the same period, we identified 11,114 individual patients in the Patient Registry and the Cause of Death Registry with registrations related to cardiac arrest. Of these patients, 3469 were already registered in NorCAR, leaving 7645 patients for manual validation. Some patients were controlled for multiple episodes. From the patient records, we eventually included 344 (4%) of the 7645 patients. The case completeness in NorCAR was 96% before the validation. The details from each registry are described in figure 1. This project resulted in an increase from 57 to 59 OHCA per 100,000 inhabitants per year and an increase in the survival incidence from 7.3 to 7.9 per 100,000 inhabitants. There was a decrease in the rate of missing patient inclusions during the study period (*p* < 0.001) (Figure 2).

We found most new cases in the case assessment from the Patient Registry with the diagnose code «I46.0 Cardiac arrest with successful resuscitation» and from the Cause of Death Registry in the category “Underlying cause of death, cardiac arrest” (135 and 122 cases, respectively) (Table 1).

The distribution of patient age, gender, presumed cardiac cause and bystander CPR was similar between the registered and missing patients. The missing patients more often had a shockable rhythm and cardiac arrest in a public place or an ambulance. Fewer missing patients had cardiac arrest witnessed by bystanders or CPR that was started or continued by EMS personnel. However, almost twice as many of the missing patients were transported to a hospital after the arrest, had sustained ROSC and were alive 24 hours and 30 days after their cardiac arrest (Table 2).

**Table 2:** Cardiac arrest characteristics for patients registered and missing in the Norwegian Cardiac Arrest Registry 2015–2017.

	Registered patients *n* = 8685 *n* (%)	Missing patients *n* = 344 *n* (%)	*p*-value	All patients *n* = 9030 *n* (%)
Age, years, median (IQR)	69 (56–79)	69 (57–79)	0.5	69 (56–79)
Female	2840 (33)	104 (30)	0.3	2944 (33)
Shockable first rhythm (VT/VF)	1843 (21)	88 (26)	0.04	1931 (21)
Presumed cardiac cause	7149 (82)	290 (84)	0.2	7449 (82)
Location of cardiac arrest^a^			< 0.001	
Residential	6280 (72)	209 (61)		6489 (72)
Public place	2292 (26)	132 (38)		2424 (27)
In the ambulance	346 (4)	26 (8)		372 (4)
Collapse witnessed by			< 0.001	
Bystander	4459 (51)	148 (43)		4607 (51)
EMS personnel	1108 (13)	55 (16)		1163 (13)
Not witnessed	4226 (49)	197 (57)		4423 (49)
Bystander CPR^b^	6007 (79)	288 (79)	0.8	6235 (79)
Response interval, minutes, median (IQR)^b^	10 (7–15)	12 (6–19)	0.4	10 (10.4)
CPR by ambulance crew	7630 (88)	245 (71)	< 0.001	7875 (87)
Transported to hospital	3120 (36)	215 (62)	< 0.001	3335 (37)
ROSC^c^	2277 (30)	138 (54)	< 0.001	2415 (30)
24-hour survival^c^	1789 (23)	132 (51)	< 0.001	1921 (24)
30-day survival^c^	1114 (15)	97 (38)	< 0.001	1211 (15)

Abbreviations: CPR, cardiopulmonary resuscitation; EMS, emergency medical services; IQR, interquartile range; ROSC, return of spontaneous circulation; VF, ventricular fibrillation; VT, ventricular tachycardia.

The numbers of missing, unknown and not applicable are included in the total of each variable.

(>2 h) are excluded from the analysis of the response interval (*n* = 4380).

a Location: residential = at home and health institutions; Public place = workplace, medical office and emergency room.

b Ambulance-witnessed cardiac arrests are excluded from this analysis.

c The denominator is patients for whom the ambulance crew has started or continued treatment, and patients defibrillated before the ambulance arrival (*n* = 7687, 258 and 7945, respectively).

All patients had complete registration for the presumed cause of arrest and high completeness for the location of the cardiac arrest. The missing patients had more missing or unknown information for witnessed status and CPR by EMS. Information about bystander CPR and sustained ROSC was similar in both groups (Table 3).

**Table 3:** The number of missing or unknown registrations for key variables for the registered and missing patients in the Norwegian Cardiac Arrest Registry 2015–2017.

	Registered patients *n* = 8685 *n* (%)	Missing patietns *n* = 345 *n* (%)
Sustained ROSC^a^	982 (13)	67 (19)
Bystander CPR	490 (6)	25 (7)
Witnessed	227 (3)	117 (43)
Location of cardiac arrest	133 (1)	4 (1)
CPR by EMS	14 (0)	19 (6)
Presumed cause of cardiac arrest	0 (0)	0 (0)

Abbreviations: CPR, cardiopulmonary resuscitation; EMS, emergency medical services; ROSC, return of spontaneous circulation.

a Sustained ROSC for patients not treated by EMS is not registered. Patients not treated by EMS are consequently excluded from this analysis.

## Discussion

Using data from the Patient Registry and the Cause of Death Registry, we found that case completeness in NorCAR was nearly 96%, and that case completeness increased from 2015 to 2017. The registered and missing patients were similar regarding age, gender, shockable rhythm and presumed cause. The missing patients more frequently achieved ROSC, were more often transported to a hospital, and had higher survival rates. The key variables were more complete for the registered patients.

The value of a registry is highly dependent on the quality of its data,16., 17. and consequently, all registries should evaluate the quality and completeness of registered cases. For in-hospital cardiac arrest registries, it is possible to use existing hospital systems, such as patient administrative data or cardiac arrest calls, for comparisons.8., 9. For OHCA patients, this is more complicated since a majority of the patients die on scene and are not admitted to hospital. Different approaches have been tried to review case inclusions of OHCA registries. Strömsöe and colleagues from the Swedish Cardiac Arrest Registry manually audited patient report forms from ambulances and found that 25% of the cases were missing. Their audit was performed on one-third of the Swedish population.^10^ Savary and colleagues used data from an EMS system and a Fire Department to assess the case completeness in a French administrative region. During four months, they found 18% missing registrations.^11^ The assessment of case completeness of NorCAR was both labour-intensive and time-consuming. A representative from the national registry travelled to the 18 health trusts to aid in the audit, with associated costs for transport, board and lodging. The visits lasted one to three days. However, our approach, linking and comparing data from the three registries, made it possible to audit NorCAR for three full years. However, a manual audit of all ambulance patient report forms might have yielded more missing patients than we were able to uncover with our choice of method.

Other studies also find that a minority of potential OHCA cases identified with administrative data are actual cases. Most of these studies have used diagnose codes suitable to identify patients surviving to hospitalization.18., 19., 20. In this study 96% of all potential cases were never confirmed as OHCA. As expected, some cases were in-hospital cardiac arrests, since data from the Patient Registry originate from hospital admissions. Some cases originated from the local branches of NorCAR that didn’t register cardiac arrest for the years we assessed the data. Further, in order to confirm missing cases from patient records, the cardiac arrest has to be documented there. Missing patient record documentation has been confirmed in a case assessment of in-hospital cardiac arrest.^8^ Additionally, a substantial proportion of the patients had repeated administrative registrations for the same cardiac arrest episode. This was typical for cases with a transfer of care between wards and hospitals. As discussed by Varmdal and colleagues, Norway has a diagnosis-related system for reimbursement, which might influence the coding processes. Besides acting as an encouragement to register all cases, it might also inspire excessive or repeated coding.^21^ Lastly, we chose to include NCMP and NCSP data for “chest compression”; these data are generated for in-hospital procedures. For the OHCA population, this signifies repeated cardiac arrest after hospital admission. Only 4% of the missing patients were found through these data. For future control of case completeness, we may consider excluding this data source.

We expected to find two-thirds of the missing inclusions from the Cause of Death Registry, corresponding to the proportion of deaths before hospital admission. However, we found two-thirds of the missing patients from in-hospital data in the Patient Registry. This might indicate that we more often miss OHCA patients surviving to hospital admission but, a more plausible explanation is that we could not identify all cases of OHCA who died in the prehospital setting from the Cause of Death Registry. Cardiac arrest is an unspecific diagnosis that can also reflect the end stage of disease or a complication before death. Unspecific diagnoses are not recommended to be used on death certificates, as they fail to give information about the causal reason for death, such as ischaemic heart disease or cardiomyopathy.^22^ Although we might have missed cases from the Cause of Death Registry, it helped us identify one-third of the missing patients we would not have been able to locate from registries based on in-hospital data.

The missing patients had more missing and unknown key variables. This was most likely because the missing patients did not have the dedicated paper form ordinarily used when entering cases in NorCAR. The data processors had to rely on the available EMS and hospital records to register the missing cases. Strömsöe and colleagues also found differences in the cardiac arrest characteristics between the registered and missing patients.^10^ Much of these differences might be attributed to the circumstances allowing registration in either the Patient Registry, with survival to hospital admission, or the Cause of Death Registry with death as the result after the cardiac arrest. The missing patients had better survival rates than the registered patients, and two-thirds were identified from the Patient Registry. Because survival rate is so dependent on both nominator (survivors) and denominator (those treated), the small number of patients we found in case assessment did not change overall survival rate in the registry (15%). However, in relation to the population at risk, the 97 extra survivors found in the study period of 3 years, increased incidence of survival from 7.3 to 7.9 per 100,000 inhabitants per year.

NorCAR focuses on education and feedback to the data processors, which might explain the high and increasing case completeness compared to other registries.^12^ The registry publishes and compares the incidence of cardiac arrest between areas. NorCAR has initiated projects comparing and improving methods for data capture between the areas with the lowest and highest incidence. After this case-control, NorCAR encouraged EMCCs to establish data processors in their unit. In addition to identifying cases from EMS personnel, this allows identifying cases from EMCCs after telephone-guided CPR. Another approach that might aid future registration and case assessments is the ongoing introduction of electronic patient reports in Norwegian prehospital services. Hopefully, electronic registration will make registration, reporting, data search and collection more manageable.

Due to the high level of case completeness, the influence of retrospective registrations on the total OHCA population was too small to alter the overall results. However, the degree of missing key variables in the new cases demonstrates the importance of a continued focus on prospective registration and feedback to ensure the high quality and validity of data in a registry.

## Strengths and Limitations

Personal identification numbers are a strength that allowed us to identify and follow all patients in Norway. They also allowed the linkage of data between different national registers that cover the entire population.

To have a complete assessment of case inclusions, the sources for comparison also need to be complete, including the patient journal systems. Due to missing an overlap of relevant patients in NorCAR registered in the Cause of Death Registry (48%) and the Patient Registry (92%), this study might be biased from missing episodes or different classifications from the latter two registries. NorCAR register patients that have received resuscitation efforts. Data from the Patient Registry and the Cause of Death Registry may consist of cases where no resuscitation was performed, where the cardiac arrest was a past diagnosis and remained supplementary for follow-up visits, or the treating physician judged the cause of death to be cardiac arrest. This might be reflected in the missing overlap between the registries.

Dispatch and ambulance records were not reviewed for the cause of missed inclusion. Some factors were associated with missing registrations, as shown in Table 2.

## Conclusion

This study provides a novel approach to examining an OHCA registry’s case completeness, using linkage with a registry for hospital administrative data and a cause of death registry. This method allows control of both patients who die in the prehospital setting and patients admitted to hospitals.

NorCAR has high case completeness, with a decreasing trend of missing patients every year. Even with some differences in patient characteristics between registered and missing patients, the high case completeness indicates that data from the registry are representative of OHCA in Norway and that the registry is a good source for quality improvement and research projects.

## Conflict of interest statement

The authors report no conflicts of interest.
